# Epilepsy Caused by the Ascaris Lumbricoides

**Published:** 1861-09

**Authors:** M. M. Eaton

**Affiliations:** Peoria


### ORIGINAL COMMUNICATIONS.

### EPILEPSY CAUSED BY THE ASCARIS LUMBRICOIDES.

By M. M. EATON, M.D., of Peoria.

Having had the care of a few cases of Epilepsy in the Chicago City Hospital, under the direction of Prof. Brainard, the last of 1859 and first of 1860, my mind was called to notice the great variety of causes producing, and the multiplicity of remedies found useful in different cases.

It was, therefore, with a degree of reluctance that I undertook the treatment of S. S., who called me Aug. 25th, 1861. He was about 30 years of age, unmarried, and a native of Massachusetts.

His brother and mother stated that he had been subject to fits for nine years; that they commenced mildly, but had steadily increased; that for four years he had had them two or three times a week, often as many times a day; and had only once in that time been free from them so long as three weeks.

They said "he had been doctored by nine different *medical* men, and had used almost all the newspaper remedies for Epilepsy. He had been been a school teacher, and had studied law two years previously to his fits getting so bad; but now he was weak, and did not perform any labor."

He looked careworn and pale, and there was a vacant stare in his eye, unpleasant to look upon; was very irritable; pulse weak.

He said he wanted to eat; but that when he did so, he felt a rising like a ball in his throat, so he could swallow but little; still he felt the gnawing of hunger in his stomach, which he was never able to satisfy.

On inquiry I ascertained that he had been twice subjected to a thorough mercurial treatment; had been bled repeatedly; had used almost all the *Veg.* and *Min.* tonics, as well as a great variety of antispasmodics and antiperiodics; and, so far as I could learn, I thought these remedies had been applied with a skillful hand.

I examined the cranium, but could find no depression, although they told me that he had, when a small boy, received a severe blow from the kick of a horse on his head.

I told them I would think of his case, and requested to be called if he took another fit, that I might see him in one, and be better able to judge of his case.

The next day he was attacked, and I, happening not to be far off, was soon by his side, and after witnessing his appearance and hearing his brother's description of the onset of the attack, I was convinced that my man had the pure, unfeigned Epilepsy, the very *grand mal* of the French.

The paroxysm lasted this time near twenty minutes; he revived, seemed rational, and then fell asleep and slept over an hour. This, I was told, was his usual custom.

On inquiry I found he was troubled with itching of the nose and anus; that his bowels were often hard and painful; and that when a small lad he had been troubled with worms.

After some reflection, I called the next day, and told him and his friends that I thought he was troubled with worms, and would give him some medicine for them. Ordered—

℞—Ol Terabin,

Ol Ricmi aa f℥ ij,

Alcohol f℥ j,

Es Cin gtt. xxx M.

Sig.—Take a large spoonful in milk after eating, three times a day. Ordered him to take but little food.

Aug. 28th—Took the medicine as directed.

Aug. 29th—Ordered and gave Ol. Ricini. ℥ j.

Aug. 30th—Found that the previous day and evening he had had copious discharges of worms, and on examination I

found them to be of the variety called the Ascaris Lumbricoides; the dejections being in amount about one quart, mostly composed of worms, nearly all of which were dead.

Ordered him to take the same medicine as on the 28th; followed the next morning by castor oil.

Sept. 1st—Found he had had a slight fit the day before, and since I saw him had been relieved of over a quart of offensive matter *per anum*, looking like a jelly of worms.

He was then very weak, and affected with strangury. I introduced the catheter and relieved him of that, and ordered him to take ℥ss of the castor oil. Saw him at 2 P. M.; found his dejections contained but slight traces of worms; ordered beef tea to be given, and teaspoonful of the following solution in water every four hours:

℞—Quinia Sulph. gr. x,

Morph. Sulph. gr. ss,

Alcohol ℥ iv,

Es. Wint. gtt. l x M.

Continued this a week and then gave the Tr. Ferri. Muriat gtt. x three times a day for a week longer. Then gave him the following pills to take three Mondays and Wednesdays for 4 or 5 weeks:

Qui. Sulph., gr. xx.
Strychnia, gr. ss.
Ferri Carb., gr. xxx. M. Ft. Pill No. xxx.

Oct. 1st—Saw my patient and learned he had followed my directions, and had had no more fits; that the itching of the nose and anus was gone and that his food went to the right place now and satisfied his hunger; there was no more of that rising in his throat he had complained of before.

Feb. 10th, 1861—When in Chicago, at Rush College, I received a letter from a friend of his, stating he had been afflicted with no more fits, but was then and had been teaching a writing school with good success.

In June last, I learned he was well and had gone to Woodford Co. to work on a farm.

